# Optimal Chimeric Antigen Receptor (CAR)-mRNA for Transient CAR T Cell Generation

**DOI:** 10.3390/ijms26030965

**Published:** 2025-01-23

**Authors:** Reni Kitte, Robert Serfling, Ulrich Blache, Claudius Seitz, Selina Schrader, Ulrike Köhl, Stephan Fricke, Christian Bär, U. Sandy Tretbar

**Affiliations:** 1 Fraunhofer Institute for Cell Therapy and Immunology (IZI), Perlickstr. 1, 04103 Leipzig, Germany; reni.kitte@izi.fraunhofer.de (R.K.); robert.serfling@izi.fraunhofer.de (R.S.); ulrich.blache@izi.fraunhofer.de (U.B.); ulrike.koehl@izi.fraunhofer.de (U.K.); stephan.fricke@izi.fraunhofer.de (S.F.); 2 Fraunhofer Cluster of Excellence Immune-Mediated Diseases (CIMD), Perlickstr. 1, 04103 Leipzig, Germany; 3 Fraunhofer Institute for Toxicology and Experimental Medicine (ITEM), Inhoffenstraße 7, 38124 Braunschweig, Germany; claudius.seitz@item.fraunhofer.de (C.S.); selina.martina.schrader@item.fraunhofer.de (S.S.); 4 Institute of Clinical Immunology, Medical Faculty, University of Leipzig, Johannisallee 30, 04103 Leipzig, Germany; 5 Medicine Campus MEDiC, Technical University of Dresden, Klinikum Chemnitz gGmbH, 09116 Chemnitz, Germany; 6 Fraunhofer Institute for Toxicology and Experimental Medicine (ITEM), Nikolai-Fuchs-Straße 1, 30625 Hannover, Germany; christian.baer@item.fraunhofer.de; 7 Fraunhofer Cluster of Excellence Immune-Mediated Diseases (CIMD), Nikolai-Fuchs-Straße 1, 30625 Hannover, Germany; 8 Institute of Molecular and Translational Therapeutic Strategies, Hannover Medical School, Carl-Neuberg-Str. 1, 30625 Hannover, Germany

**Keywords:** transient CAR T cells, T cell engineering, mRNA design, in vitro transcription, dsRNA, immunogenicity

## Abstract

Genetically modified T lymphocytes expressing chimeric antigen receptors (CARs) are becoming increasingly important in the treatment of hematologic malignancies and are also intensively being investigated for other diseases such as autoimmune disorders and HIV. Current CAR T cell therapies predominantly use viral transduction methods which, despite their efficacy, raise safety concerns related to genomic integration and potentially associated malignancies as well as labor- and cost-intensive manufacturing. Therefore, non-viral gene transfer methods, especially mRNA-based approaches, have attracted research interest due to their transient modification and enhanced safety profile. In this study, the optimization of CAR-mRNA for T cell applications is investigated, focusing on the impact of mRNA modifications, in vitro transcription protocols, and purification techniques on the translation efficiency and immunogenicity of mRNA. Furthermore, the refined CAR-mRNA was used to generate transient CAR T cells from acute myeloid leukemia patient samples, demonstrating efficacy in vitro and proof-of-concept for clinically relevant settings. These results highlight the potential of optimized mRNA to produce transient and safe CAR T cells.

## 1. Introduction

Genetically modified T lymphocytes expressing a chimeric antigen receptor (CAR) have emerged as a significant component in the treatment landscape for various hematological malignancies [1], with their indications progressively expanding to non-cancerous diseases, such as autoimmune diseases [2,3,4] and HIV infections (e.g., NCT04648046 [5], NCT05077527, NCT03617198). All approved CAR T cell therapies and most of the therapies used in clinical trials rely on the viral transduction of T cells using gammaretroviral or lentiviral vectors for genetic modification due to their capability in terms of reaching high transduction rates and stable CAR expression [1,6]. Although viral transduction is well functioning and despite its widespread use, it is accompanied by a time- and cost-intensive CAR T cell manufacturing process and safety concerns arising from the genomic integration of the virus [7]. Although the frequency was very low, the FDA has determined a risk of secondary malignancies such as myeloid and T cell neoplasms for patients receiving CAR T cells [8,9] suggesting a potential contribution of CAR gene integration to the development of T cell malignancies [10]. Due to these concerns and limitations, non-viral gene transfer methods have increasingly become the focus of research, particularly for the treatment of autoimmune diseases, which require a higher safety level for the CAR T cell product compared to malignant diseases [11,12].

One non-viral alternative is the transfer of CAR-encoding mRNA into T cells, as it offers a non-integrating and transient T cell modification, providing an improved safety profile and the controllability of CAR T cell therapy [13,14,15,16]. Furthermore, mRNA can be produced by in vitro transcription (IVT) in a robust and cost-effective manner and in a short time [17]. mRNA-engineered CAR T cells have already been investigated in different preclinical models of hematological and solid malignancies as well as in several clinical trials (reviewed by Soundara Rajan et al. [14]). More recently, mRNA-CAR T cells addressing B cell-targets were also clinically tested as a treatment option for autoimmune diseases, e.g., systemic lupus erythematosus and myasthenia gravis (NCT04146051) and reported as well tolerated by showing a clinically meaningful decrease in disease severity [2,18].

Although IVT-mRNA is being considered as a promising non-viral vector for CAR T cell generation, there are several production and design parameters that can affect mRNA function and stability [19], and thus potentially determine the success of mRNA-based CAR T cell therapy. For instance, one major issue of IVT-mRNA in most therapeutic applications is its potential immunogenicity arising from the production of double-stranded RNA (dsRNA) as a by-product of IVT [17,20,21,22,23]. DsRNA and other possible immunogenic transcription products can be recognized by both endosomal innate immune receptors, such as Toll-like receptor 3 (TLR3), TLR7, and TLR8 [24,25], as well as various cytoplasmic innate immune receptors, mainly retinoic acid-inducible gene 1 protein (RIG-1), melanoma differentiation-associated protein 5 (MDA5), protein kinase R (PKR), and 2′-5′-oligoadenylate synthase (OAS), resulting in the secretion of proinflammatory cytokines, the inhibition of mRNA translation, and the induction of mRNA degradation (reviewed by Sahin et al. [17]). Several strategies have been described to reduce the formation of dsRNA during IVT, including IVT process optimizations [22,26,27] or the usage of engineered T7 RNA polymerases [28,29] or modified nucleotides, such as pseudouridine (ψ) or 5-methylcytidine (5mC) [30,31,32,33]. N1-methylpseudouridine (m1ψ)- and m1ψ+5mC-modified mRNA have been reported to effectively evade TLR recognition and to provide up to 44-fold increased protein expression than unmodified or ψ-incorporated mRNA in various cell lines, thus being proposed as new standard in the field of mRNA-based therapeutics [34]. However, in primary macrophages, both m1ψ- and m1ψ+5mC-modified mRNAs showed comparable or reduced transgene expression compared to unmodified or ψ-mRNA [35], indicating the cell-type-dependent effects of nucleotide modifications.

In this study, the optimization of a CAR-mRNA directed against CD123 for the treatment for the dedicated use in T cells, especially for CAR T cell generation and the treatment of acute myeloid leukemia (AML), was investigated. CD123 is a promising target for AML due to its widespread expression on AML cells, making it a target antigen in various preclinical and clinical studies [36]. CD123-directed CAR T cells have demonstrated significant effectiveness in selectively killing CD123-expressing AML cell lines in vitro. However, CD123 is also present on healthy hematopoietic stem cells [36,37] and endothelial cells [38]. Targeting immature hematopoietic stem cells with CD123-directed CAR T cells has been shown to have an ablative effect on hematopoiesis in vivo [39], and there are concerns about potential damage to the endothelium [40], which poses a significant issue for CD123-directed CAR T cell therapy. Therefore, the continuous expression of CD123-directed CARs raises safety concerns that could potentially be mitigated by using transient CAR T cells. For effective transient CAR expression, common mRNA adaptations such as 5′ cap, untranslated regions (UTRs) and modified nucleotides, as well as the influence of IVT protocols and purification methods on the translation efficiency and immunogenicity of the respective mRNAs were analyzed. The optimized CAR-mRNA was used in a proof-of-concept experiment to generate transient CAR T cells from AML patient samples and its efficacy was successfully demonstrated in a more clinically relevant setting.

## 2. Results

### 2.1. Essential Structures of IVT-mRNA

**Figure 1:**
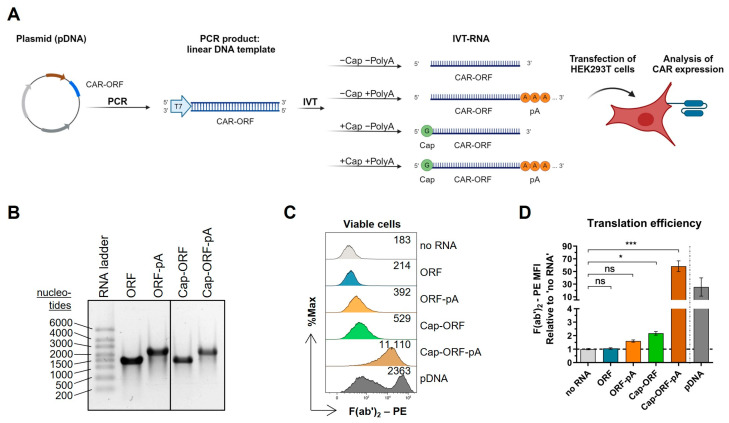
Basic requirements for the generation of functional CAR-mRNA. (**A**) Workflow. PCR was performed from a lentiviral transfer plasmid (plasmid DNA, pDNA) to generate the linear DNA template of the CAR-open reading frame (CAR-ORF) with the T7 promotor region. In vitro transcription (IVT) was carried out either in the absence (−Cap) or presence of an anti-reverse cap analog (+Cap), and either without (−PolyA) or with subsequent polyadenylation (+PolyA). The functionality of the resulting IVT-RNA variants was tested by the transfection of HEK293T cells and analysis of CAR expression. Illustration was created with BioRender.com. (**B**) Representative agarose gel image of the IVT-RNA variants: non-capped, non-polyadenylated RNA (ORF); non-capped, polyadenylated RNA (ORF-pA); capped, non-polyadenylated RNA (Cap-ORF); capped, polyadenylated RNA (Cap-ORF-pA). (**C**) Representative histograms of flow cytometric CAR expression analysis of HEK293T cells 24 h after transfection with no RNA, RNA variants from (**B**), as well as with pDNA used as positive control for transfection. Values indicate F(ab’)_2_–phycoerythrin (PE) median fluorescence intensity (MFI) as an indicator of CAR intensity on the cell surface to demonstrate the translation efficiency of RNA variants. (**D**) Translation efficiency relative to ‘no RNA’ cells. Values are presented as mean ± SEM of n = 5 independent experiments performed in technical duplicates. ns: not significant; * *p* < 0.05; *** *p* < 0.001, Kruskal–Wallis test with corrected Dunn’s multiple comparisons test.

To evaluate the essential minimal features required for the functionality of an anti-CD123 CAR-mRNA, additional steps including co-transcriptional 5′ capping with an anti-reverse cap analog (ARCA) and/or subsequent post-transcriptional polyadenylation were carried out. As a result, different IVT-RNA variants were obtained: ORF (open reading frame of the CAR, without a 5′ cap and poly(A) tail), Cap-ORF (with a 5′ cap but without a poly(A) tail), ORF-pA (with a poly(A) tail but without the 5′ cap), and Cap-ORF-pA (with both the cap and poly(A) tail (Figure 1A)). Agarose gel electrophoresis analysis revealed full-length RNA of about 1500 nucleotides (expected: 1458 nucleotides of ORF and Cap-ORF RNAs) as well as extension of polyadenylated (ORF-pA, Cap-ORF-pA) compared to non-polyadenylated RNA (ORF, Cap-ORF) (Figure 1B). To analyze mRNA functionality, HEK293T cells were transfected with the same molar mass of each IVT-RNA variant. The translation efficiency of IVT-RNA was evaluated by analyzing CAR intensity on the cell surface indicated by the F(ab’)_2_–phycoerythrin (PE) median fluorescence intensity (MFI) (Figure 1C). Cells transfected with ORF did not display any CAR expression, indicating no translation. Cells transfected with ORF-pA or Cap-ORF showed a slight enhancement of CAR expression. The strongest CAR expression was observed in cells transfected with Cap-ORF-pA (about a 60-fold increase compared to no RNA and ORF), indicating high translation efficiency (Figure 1D). These results demonstrate that the presence of both the 5′ cap and poly(A) tail is essential for IVT-CAR-mRNA functionality in cells.

### 2.2. Improvement of CAR Expression Using CleanCap AG (3′ OMe) as 5′ Cap Analog

**Figure 2:**
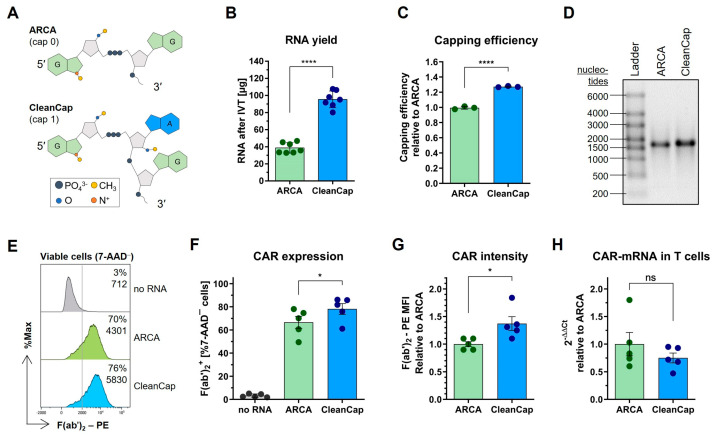
Comparison of classical anti-reverse cap analog (ARCA) with CleanCap AG (3′ OMe) (CleanCap) in CAR-mRNA. (**A**) Simplified chemical formulas, which present the main differences in the chemical structure. The illustration was adapted from [35] under the Creative Commons CC-BY license. (**B**) Produced RNA quantity after IVT to indicate the transcription efficiency. (**C**) Capping efficiency normalized to ARCA-RNA. (**D**) Representative agarose gel image of ARCA- and CleanCap-mRNAs after polyadenylation. (**E**) Representative histograms of the flow cytometric CAR expression analysis of viable (7-AAD^−^) primary T cells 24 h after transfection via electroporation with no RNA or with ARCA- or CleanCap-mRNA. A vertical line defines the border between CAR^−^ (left side) and CAR^+^ cells (right side). Values indicate the percent of CAR^+^ cells (top) and F(ab’)_2_–phycoerythrin (PE) median fluorescence intensity (MFI) as an indicator of CAR intensity on the cell surface (bottom). (**F**) Percent of CAR^+^ T cells 24 h post-transfection. (**G**) CAR intensity on the T cell surface relative to cells that received ARCA-mRNA 24 h post-transfection. (**H**) Quantification of transferred CAR-mRNA of transfected T cells 24 h after transfection relative to ARCA-mRNA quantity. (**B**,**C**) Bars indicate mean ± SD of RNA produced in (**B**) n = 7 or (**C**) n = 3 independent IVT reactions presented by dots. (**F**–**H**) Bars indicate mean ± SEM of n = 5 independent experiments performed with T cells from five different donors presented by the dots. ns: not significant; * *p* < 0.05; **** *p* < 0.0001, (**B**,**C**) unpaired *t*-test or (**F**–**H**) paired *t*-test.

Anti-reverse cap analogs (ARCAs) are widely used as cap analogs during IVT for co-transcriptional capping, as the incorporation of the cap into RNA in the wrong orientation is prevented by a methyl group on the 3′O position of 5′ guanosine leading to an improved translation [41]. The “classical” first-generation ARCA results in a cap 0 structure. However, cap 1 structures, which carry an additional methyl group at the 2′O position of the first transcribed nucleotide, are more prevalent in higher eukaryotes such as humans, compared to cap 0 structures [42]. Recently, an ARCA variant that generates a cap 1 structure, termed CleanCap AG (3′ OMe), was reported to yield a higher capping efficiency than the cap 0 ARCA variant [43]. Therefore, we investigated two versions of ARCA side-by-side: a cap 0 structure represented by the classical ARCA (referred to as ARCA) and a cap 1 structure represented by CleanCap AG (3′ OMe) (referred to as CleanCap) (Figure 2A). 

The synthesis of RNA by IVT with co-transcriptional CleanCap capping resulted in an about 2.5-fold larger RNA quantity (Figure 2B) as well as a significantly higher proportion of capped RNA (Figure 2C) compared to that obtained with ARCA capping. After polyadenylation and the confirmation of full-length mRNA (Figure 2D), primary human T cells were transfected to analyze the translatability of ARCA- and CleanCap-mRNAs. CleanCap-mRNA led to a significantly higher proportion of CAR^+^ cells and an increased CAR intensity on the T cell surface compared to ARCA-mRNA (Figure 2E–G). These observations were not a result of different transfection efficiencies, as the quantity of CAR-mRNA inside the T cells was comparable (Figure 2H). These results suggest that the enhanced CAR expression in CleanCap-mRNA-transfected T cells is due to the larger amount of capped RNA owed to the increased capping efficiency during IVT. 

### 2.3. Improvement of CAR Expression by Untranslated Regions

**Figure 3:**
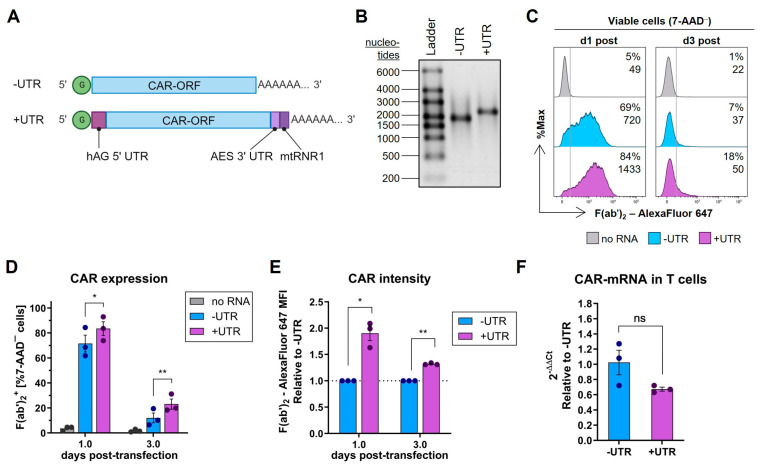
Impact of 5′ and 3′ untranslated regions (UTRs) in CAR-mRNA. (**A**) Structures of CAR-mRNA without UTRs (-UTR) and with UTRs (+UTR) using the 5′ UTR of human alpha globin (hAG) and both the 136 nt core element of amino-terminal enhancer of split (AES)-mRNA and mitochondrially encoded 12S rRNA (mtRNR1) positions 112–250 as joint 3′ UTR. Besides UTRs, CAR-open reading frame (CAR-ORF) was flanked by CleanCap as the 5′ cap structure (green “G”) and the 3′ poly(A) tail. Illustration was created with BioRender.com. (**B**) Representative agarose gel image of CAR-mRNAs after polyadenylation. (**C**) Representative histograms of flow cytometric CAR expression analysis of viable (7-AAD^−^) primary T cells one (d1 post) and three days (d3 post) after transfection via lipid nanoparticles with no RNA or 1.0 pmol/10^6^ cells of -UTR-mRNA or +UTR-mRNA. The vertical line defines the border between CAR^−^ (left side) and CAR^+^ cells (right side). Values indicate percent of CAR^+^ cells (top) and F(ab’)_2_–AlexaFluor 647 median fluorescence intensity (MFI) as an indicator of CAR intensity on the cell surface (bottom). (**D**) Percent of CAR^+^ T cells. (**E**) CAR intensity on the T cell surface relative to cells that received -UTR-mRNA. (**F**) Quantification of transferred CAR-mRNA one day after T cell transfection relative to CAR-mRNA quantity of cells that were transfected with -UTR-mRNA. (**D**–**F**) Bars and values indicate mean ± SEM of n = 3 independent experiments performed with T cells from three different donors presented by dots. ns: not significant; * *p* < 0.05; ** *p* < 0.01. (**D**,**E**) Multiple paired *t*-tests corrected for multiple comparisons using Holm–Sidak method or (**F**) paired *t*-test.

As 5′ and 3′ UTRs are known key regulators of mRNA translatability and stability [17], CAR-mRNA without UTRs was compared to CAR-mRNA with UTRs regarding their translation in primary T cells. Therefore, the 5′ UTR of human alpha globin (hAG) was selected as 5′ UTR sequence and both the 136 nt 3′ UTR core element of AES-mRNA and mtRNR1 positions 112–250 were selected as joint 3′ UTR motive (Figure 3A), since these combinations have already been reported to provide the highest mRNA half-life and to promote the highest transgene expression in several other cell types [44]. mRNAs, each with a full N^1^-methylpseudouridine (m1ψ) modification, were prepared and analyzed using agarose gel electrophoresis showing a clearly longer mRNA with UTRs (mRNA+UTRs) compared to mRNA without UTRs (mRNA-UTRs) (expected elongation by 315 nucleotides) (Figure 3B), confirming the presence of the UTRs. 

To investigate the impact of the selected UTRs in m1ψ-CAR-mRNA on the translation efficiency in T cells, primary T cells were transfected with mRNA-UTRs and mRNA+UTRs. CAR expression and intensity were analyzed on day 1 and again on day 3 post-transfection to see whether the effects were maintained over time. On both analysis days, mRNA+UTRs resulted in significantly larger proportions of CAR^+^ cells (Figure 3C,D), including 1.9-fold and 1.3-fold increased CAR intensities on the T cell surface on day 1 and on day 3, respectively (Figure 3E). The quantity of mRNA+UTRs in T cells was slightly lower but not significantly different from that of mRNA-UTRs (Figure 3F), suggesting that the enhanced CAR intensity with mRNA+UTRs may be due to its improved translation efficiency of mRNA containing the 5′ hAG UTR and 3′ AES/mtRNR1 UTR compared to mRNA without these motifs. 

### 2.4. Reduction in mRNA Immunogenicity by N^1^-Methylpseudouridine (m1ψ)

**Figure 4:**
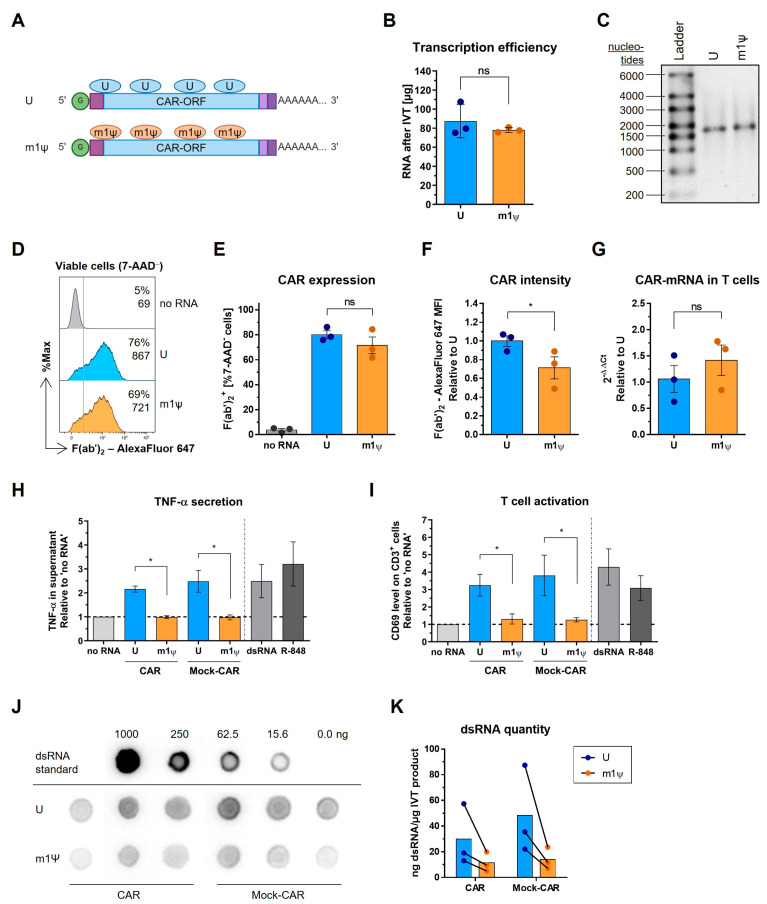
Impact of N^1^-methylpseudouridine (m1ψ) in CAR-mRNA. (**A**) Structure of unmodified CAR-mRNA with uridine (U) and N^1^-methylpseudouridine (m1ψ)-modified CAR-mRNA. Both variants comprise the CAR-open reading frame (CAR-ORF), the 5′ hAG UTR and 3′ AES/mtRNR1 UTR (violet), CleanCap as 5′ cap structure (green “G”), and the 3′ poly(A) tail. Illustration was created with BioRender.com. (**B**) Produced RNA amount during in vitro transcription (IVT) to indicate transcription efficiency. (**C**) Representative agarose gel image of CAR-mRNAs after polyadenylation. (**D**) Representative histograms of flow cytometric CAR expression analysis of viable (7-AAD^−^) primary T cells 24 h after transfection via lipid nanoparticles with no RNA or 1.0 µg/10^6^ cells of U- or m1ψ-mRNA. Vertical line defines the border between CAR^−^ (left side) and CAR^+^ cells (right side). Values indicate the percent of CAR^+^ cells (top) and F(ab’)_2_–AlexaFluor 647 median fluorescence intensity (MFI) as an indicator of CAR intensity on the cell surface (bottom). (**E**) Percent of CAR^+^ T cells. (**F**) CAR intensity on the T cell surface relative to cells that received U-mRNA. (**G**) Quantification of CAR-mRNA in transfected T cells 24 h after transfection relative to CAR-mRNA quantity of cells transfected with U-mRNA. (**H**,**I**) Human peripheral blood mononuclear cells (PBMCs) were transfected with U-mRNA and m1ψ-mRNA and 24 h later analyzed regarding (**H**) tumor necrosis factor-α (TNF-α) secretion determined in the supernatant via ELISA and (**I**) CD69 expression levels on CD3^+^ cells determined by flow cytometry to analyze mRNA immunogenicity. Double-stranded RNA (dsRNA) and resiquimod (R-848) were used as positive controls. (**J**) Dot blot for quantification of dsRNA in IVT product. Different amounts of dsRNA were used as standard (top) and 1000 ng of IVT product prepared in three independent reactions (bottom) were applied. (**K**) Densitometrically quantified dsRNA content in IVT products. (**B**) Bars indicate mean ± SD of RNA produced in n = 3 independent IVT reactions presented by dots. (**E**–**I**) Bars indicate mean ± SEM of n = 3 independent experiments performed with cells from three different donors. (**K**) Bars indicate mean of n = 3 independent IVT reactions indicated by dots to produce U-RNA and m1ψ-RNA side-by-side. Respective pairs are connected by lines. ns: not significant; * *p* < 0.05, (**B**) unpaired *t*-test with Welch’s correction, (**E**–**G**) paired *t*-test, (**H**,**I**) one-way ANOVA (matched values) corrected for multiple comparisons using Holm–Sidak method.

Since nucleotide modifications are known for improving protein expression by enhancing mRNA stability and reducing mRNA immunogenicity, the effect of m1ψ-, 5mC, and m1ψ+5mC-modifications, which had been suggested for the usage in therapeutic applications [34,45], was evaluated. First, mRNA encoding enhanced green fluorescent protein (EGFP) containing these selected modifications was produced. After the transfection of HEK293T cells, the highest EGFP fluorescence intensity was derived from m1ψ-mRNA, while the reduction in EGFP fluorescence intensities by at least half resulted from 5mC- and m1ψ+5mC-mRNA compared to that from unmodified mRNA (Figure S1A). As T cells represent the target cells of CAR-mRNA for CAR T cell generation, the translation of modified EGFP-mRNAs was also analyzed in primary T cells showing the strongest EGFP mean fluorescence intensity (MFI) produced by unmodified mRNA, followed by m1ψ-mRNA, (reduction by about 25%), then by 5mC-mRNA (reduction by about 60%), and last by m1ψ+5mC-modified mRNA (reduction by 88%) (Figure S1B,C). Due to the great loss of EGFP MFI by >50%, indicating clearly diminished translation efficiency, 5mC-single-modification and m1ψ+5mC-double-modification were excluded, while m1ψ-single-modification was subsequently investigated in CAR-mRNA, which was generated by the complete substitution of UTP by m1ψ in IVT (Figure 4A). A comparable RNA amount to unmodified RNA (uridine-RNA, U-RNA) was produced (Figure 4B), and only full-length mRNA was observed after agarose gel electrophoresis (Figure 4C), indicating that m1ψ did not affect the transcription reaction. After primary T cells transfection, the proportion of CAR^+^ cells was slightly but not significantly decreased in T cells that received m1ψ-mRNA (Figure 4D,E). Moreover, CAR intensity on m1ψ-mRNA-T cells was significantly lower by about 30% on average compared to that on U-mRNA-T cells (Figure 4F), although an approximately 1.4-fold greater CAR-mRNA quantity was detected (Figure 4G), again showing an impaired translation of m1ψ-mRNA in T cells compared to U-mRNA. 

The immunogenicity of U-mRNA and m1ψ-mRNA was analyzed in the RNA immunogenicity assay by monitoring CD69 as early activation marker for T cells as well as tumor necrosis factor alpha (TNF-α) secretion. Besides modified and unmodified CAR-mRNA, a Mock-CAR construct with CAR signaling deficiency due to a lack of the co-stimulatory and CD3ζ signaling domains were tested, to rule out CAR-mediated immune cell activation as a result of tonic signaling. Double-stranded RNA (dsRNA) and resiquimod (R-848), an agonist of TLR7 and TLR8, were used as positive controls for immune activation. Irrespective of whether CAR- or Mock-CAR-mRNA were used, U-mRNA elicited an at least 2-fold increased TNF-α secretion compared to no RNA, which was significantly higher than detected for m1ψ-mRNA causing no altered TNF-α secretion (Figure 4H). Furthermore, dsRNA and R-848, U-mRNA led to an increase in CD69 levels on CD3^+^ cells by about 3.2- (CAR) to 3.8-fold (Mock-CAR), indicating T cell activation, which was only slightly affected by m1ψ-mRNA with an increase of up to 1.3-fold (Figure 4I). Both significantly reduced TNF-α secretion and T cell activation of PBMCs indicate a lower extend of immune responses towards m1ψ-mRNA compared to U-mRNA. As the lower immunogenicity of m1ψ-mRNA could be a result from a lower dsRNA amount in the IVT product, dsRNA was determined by dot blot analysis (Figure 4J). M1ψ-modification led to a reduction in dsRNA quantity by 2.6 (CAR) or 3.4 (Mock-CAR) on average, ending up in about 11.5 ng dsRNA/µg m1ψ-IVT product compared to 30.0 ng dsRNA/µg U-IVT product for CAR-mRNA and 14.1 ng dsRNA/µg m1ψ-IVT product compared to 48.3 ng dsRNA/µg U-IVT product for Mock-CAR-mRNA, respectively (Figure 4K). 

Altogether, m1ψ modification in CAR-mRNA resulted in a purer product due to less generation of dsRNA contaminant, which reduces the immunogenicity of mRNA and balances the translation effect in T cells which was only slightly impaired.

### 2.5. Improvement of CAR Expression by Choice of DNA Template

**Figure 5:**
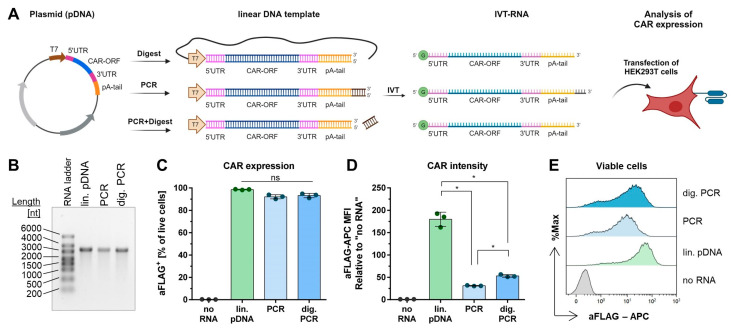
Impact of template preparation on CAR expression. (**A**) Workflow. A linear DNA template was generated from a plasmid encoding a T7 promoter, 5′ and 3′ UTRs, the CAR-open reading frame (CAR-ORF), and a poly(A) tail. The plasmid is either linearized directly downstream of the poly(A) tail by restriction digestion (lin. pDNA) or produced by PCR with primers annealing to the T7 promoter and downstream of the poly(A) region. The PCR product was either used directly as template (PCR) or digested with the same restriction enzyme used for plasmid linearization (dig. PCR). IVT was performed after spin column-based purification of the respective DNA template, and the functionality of the resulting IVT-RNA variants was tested by the transfection of HEK293T cells and an analysis of CAR expression. The figure was created using BioRender.com. (**B**) Representative agarose gel image of CAR-mRNAs produced from the three different template forms (lin. pDNA, PCR or dig. PCR). (**C**) Percentage of CAR^+^ HEK293T cells 24 h after transfection. (**D**) CAR intensity on the HEK293T cell surface 24 h after transfection relative to cells that received no mRNA. (**E**) Representative histograms of the flow cytometric CAR expression analysis of live HEK293T cells (7AAD^−^) 24 h after transfection with CAR-mRNA produced from different template formats. (**C**,**D**) Bars indicate mean ± SEM of n = 3 independent transfections of HEK293T cells performed in technical duplicates. Individual means of each transfection presented by dots. ns: not significant; * *p* < 0.05, one-way ANOVA (matched values) corrected for multiple comparisons using Holm–Sidak method.

Linearized plasmid DNA and PCR products are the most widely used and most scalable forms of IVT templates. For this reason, the influence of the respective template form on the final expression of the CAR was investigated using a CAR plasmid encoding the T7 promoter, a 5′- and 3′-UTR, the CAR gene, and a poly(A) tail. To generate the linear IVT template, the plasmid was either digested directly downstream of the poly(A) tail or a PCR product was generated with primers annealing to the T7 promoter and downstream of the poly(A) tail leading to additional nucleotides at the 3′-end. To assess the impact of additional nucleotides downstream of the poly(A) tail, the PCR product was either digested or left undigested (Figure 5A).

All IVT reactions yielded CAR-mRNA with comparable integrity and purity (Figure 5B).

The transfection of HEK293T cells with the generated CAR-mRNAs showed no significant differences in transfection efficiency, as >90% of all cells expressed the CAR for all mRNAs tested (Figure 5C).

Interestingly, the different template forms led to strong differences in the CAR expression levels on the cell surface (Figure 5D,E).

HEK293T cells transfected with IVT product from linearized plasmid had a 3.4-fold higher relative MFI than cells transfected with PCR-derived mRNA potentially due to incomplete digest of the PCR product. Notably, cells with an undigested PCR product and additional 3′ nucleotides downstream of the poly(A) tail as the IVT template had the lowest relative MFI, most likely due to the reduced binding of poly(A)-binding proteins during translation initiation. Nevertheless, these cells also showed a clearly detectable CAR expression. Therefore, the PCR protocol was further optimized to add a poly(A) tail without additional nucleotides, which further increased the CAR expression levels compared to mRNA from a digested PCR product (Figure S2). Taken together, these data suggest that the optimized CAR-mRNA can be expressed independently of template form, with the linearized plasmid or optimized PCR DNA being the recommended format.

### 2.6. Engineered RNA Polymerases for Robust In Vitro Transcription

**Figure 6:**
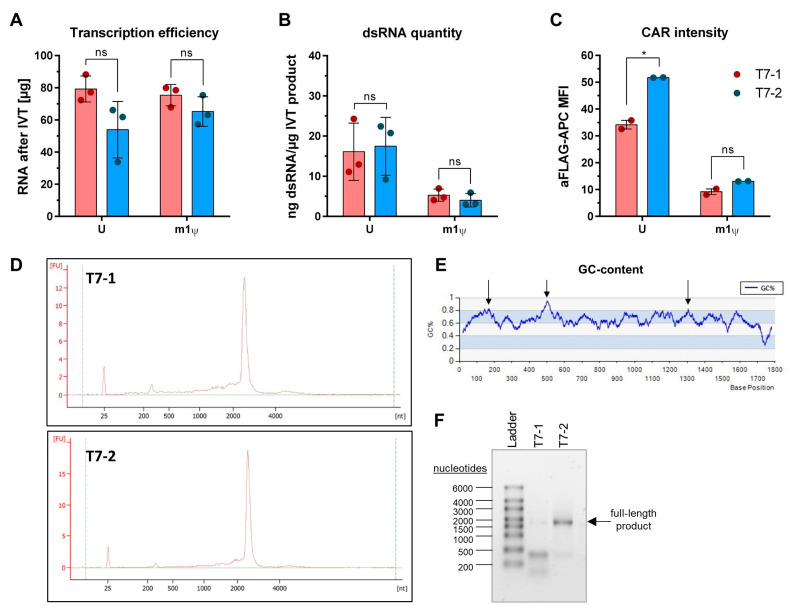
Impact of RNA polymerase on CAR-mRNA production and expression. (**A**) Average amounts of CAR-mRNA with uridine (U) and N^1^-methylpseudouridine (m1ψ)-modified CAR-mRNA obtained using two different engineered variants of the T7 polymerase. (**B**) Densitometrically quantified dsRNA content in IVT products. (**C**) CAR intensity on the T cell surface displayed as the MFI of live cells (7AAD^−^). (**D**) Representative electropherograms of 100 ng of unmodified CAR-mRNAs produced with protocols T7-1 (top) and T7-2 (bottom). (**E**) GC-content analysis of challenging CAR-mRNA with an overall GC-content higher than 65%. Arrows indicate areas with a local GC-content higher than 80%. (**F**) Representative agarose gel image of challenging CAR-mRNA produced with either T7-1 or T7-2 polymerase. (**A**,**B**) Bars indicate mean ± SD of RNA produced in n = 3 independent IVT reactions presented by dots. (**C**) Bars indicate mean ± SEM from technical triplicates of n = 2 independent experiments performed with cells from two different donors. (**A**–**C**) ns: not significant; * *p* < 0.05, one-way ANOVA (matched values) corrected for multiple comparisons using the Holm–Sidak method.

With the increasing importance of RNA for therapeutic applications, the need for cost-efficient mRNA production is also growing. For this reason, we compared our already established and well-tested IVT protocol (T7-1) with a polymerase optimized for high capping efficiency (T7-2), which can reduce the cost per µg of IVT product. Both IVT protocols generated comparable amounts of standard CAR-mRNA at 20 µL reaction scale, with T7-1 achieving slightly higher overall yields (Figure 6A). This trend was observed for both U-RNA and m1ψ-RNA. The amount of dsRNA produced also did not differ significantly between the protocols tested with an average of 16.1 ng dsRNA/µg for T7-1 and 17.4 ng dsRNA/µg for T7-2 using UTP and 5.3 ng dsRNA/µg for T7-1 and 4.0 ng dsRNA/µg for T7-2 using m1ψ (Figure 6B). Interestingly, T cells transfected with unmodified CAR-mRNA from protocol T7-2 showed a significantly higher (1.5-fold) CAR expression than comparable mRNA from protocol T7-1 (Figure 6C), most likely due to fewer truncated termination fragments produced with the protocol T7-2 (Figure 6D). A key difference between the two protocols used was found when transcribing a challenging CAR template with a high GC content (>65%) and three sections with over 80% local GC content (Figure 6E). Protocol T7-1 increasingly resulted in the premature termination of transcription in areas with very high GC content and an overall low fraction of full-length RNA (Figure 6F). In contrast, protocol T7-2 produced mainly full-length mRNA with a significantly lower proportion of truncated termination products. These data highlight the importance of a robust transcription protocol and its impact on the overall success of studies using mRNA-based transient CAR T cells.

### 2.7. Impact of Purification on CAR Expression and mRNA Immunogenicity of m1ψ-Modified mRNA

**Figure 7:**
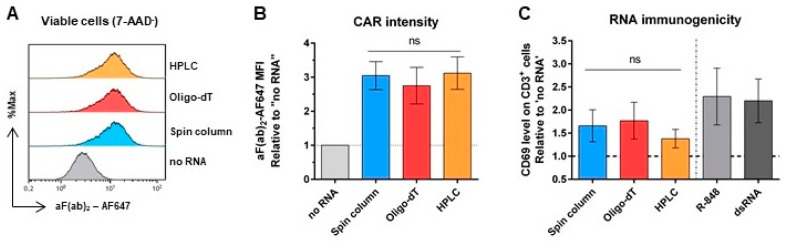
Impact of mRNA purification methods in N^1^-methylpseudouridine (m1ψ)-modified CAR-mRNA. (**A**) Representative histograms of the flow cytometric CAR expression analysis of viable (7-AAD^−^) primary T cells 24 h after transfection. (**B**) CAR intensity on the T cell surface relative to cells that received no mRNA. (**C**) Immunogenicity of mRNA indicated by CD69 expression levels on CD3^+^ cells within PBMCs using resiquimod (R-848) and double-stranded RNA (dsRNA) as positive controls. (**B**,**C**) Bars indicate mean ± SEM of n = 2 (**B**) and n = 3 (**C**) independent experiments performed with cells from three different donors. ns: not significant, one-way ANOVA (matched values) corrected for multiple comparisons using Holm–Sidak method.

To analyze whether an advanced mRNA purification method can improve the functionality and especially the quality of mRNA, which was already optimized by the use of CleanCap analog, UTRs, and nucleotide modification, m1ψ-CAR-mRNA IVT-products underwent either spin-column or more advanced purified using Oligo-dT beads or high-performance liquid chromatography (HPLC, Figure S3) and in vitro testing for their CAR expression in T cells and mRNA immunogenicity. In a direct comparison, no significant differences in the translation efficiency of the CAR-mRNA were found between the purification methods (Figure 7A,B). Furthermore, spin column-based purification, as well as both advanced purification methods led to similarly low T cell activation in comparison to the positive controls (Figure 7C), indicating that the selected purification method no longer has a major influence on mRNA immunogenicity when using optimized mRNA and the best possible IVT reaction conditions.

### 2.8. Proof of Concept for Clinical Setting by Utilising Patient Material

All mRNA-CAR T cells produced in the previous experiments were derived from the T cells of healthy blood donors, but this does not reflect the clinical situation in which CAR T cells are generated from cancer patients’ material. As a proof-of-concept study for a clinically relevant setting, CD123-directed mRNA-CAR T cells were produced from three cancer patients’ T cells in a lab scale using the optimized CAR-mRNA comprising the CleanCap as 5′ cap structure, the 5′ hAG UTR, the 3′ AES/mtRNR1 UTR, the 3′ poly(A) tail, as well as a full m1ψ-modification. Lipid nanoparticles (LNPs) were used as the mRNA delivery method recently shown by us to be superior to the state-of-the-art method, electroporation, and using T cells from healthy blood donors [46]. As CD123 represents a promising therapeutic target for CAR T cells in the treatment of AML [47,48], peripheral blood from three AML patients at initial diagnosis was selected (patient 1: acute monoblastic/monocytic leukemia; patient 2: acute promyelocytic leukemia; patient 3: AML (not further specified)). None of the patients received any type of cancer treatment at the time of blood collection. Importantly, patients’ PBMCs demonstrated extremely reduced proportions of T cells (4–10% CD3^+^) including T cell subtypes compared to healthy blood counts (48–71% CD3^+^) (Figure S4A), which affected T cell isolation and expansion. The purity of T cells derived from AML patients was much lower compared to enriched T cells from healthy donors, which could be traced back to the presence of CD33^+^ and CD123^+^ cells indicative for AML cells (Figure S4B). While enriched T cells of healthy donors started to expand on about day 3 and reached about 100-fold expansion on day 7 in average presenting the day of transfection, start of expansion of cells from AML patients was delayed by about three days (Figure S4C), most probably due to the loss of non-T cells/AML cells present at the onset of T cell culture. Thus, the timing of transfection was adapted to the delayed expansion behavior of cells from AML patients and performed on day 10 post-isolation and -activation. On that day, cells were expanded by about 72-fold on average, and T cell purity was raised to >99% with no detectable remaining AML cells (Figure S4D). 

**Figure 8:**
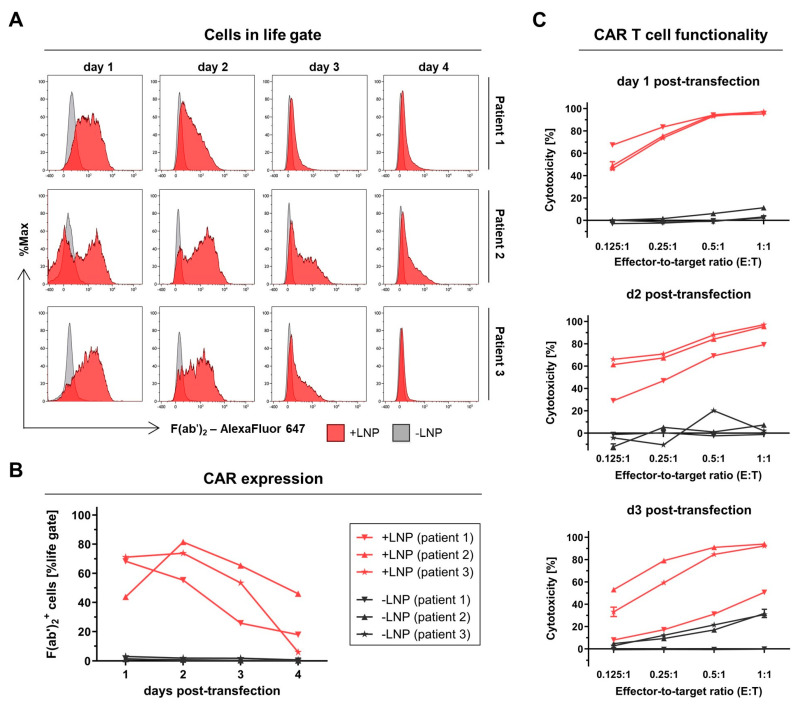
Generation and efficacy of CAR T cells derived from T cells from AML patients. After enrichment, activation, and expansion for ten days, T cells were transfected with 6 µg CAR-mRNA per 10^6^ cells using lipid nanoparticles (+LNPs, red) and analyzed using non-transfected T cells (-LNPs, grey) as negative control. (**A**) Histograms of flow cytometric CAR expression analysis from day 1 to day 4 after T cell transfection. (**B**) Percent of CAR^+^ cells indicated by F(ab’)_2_^+^ cells. (**C**) CAR T cell functionality indicated by cytotoxicity against KG-1 cells in a co-culture using different effector-to-target ratios for 24 h, determined on day 1 (top), day 2 (middle), and day 3 (bottom) post-transfection. Values are presented as mean ± SD with n = 3 technical triplicates.

After mRNA-LNP-based transfection, the CAR could be detected for up to four days (Figure 8A). In healthy T cells, CAR expression levels lasted up to 5 days when transfected with LNP [46]. While the peak CAR expression on T cells derived from patient 1 was observed on day 1 (68% F(ab’)_2_^+^), the CAR was maximally expressed on T cells from patients 2 and 3 only on day 2 post-transfection (F(ab’)_2_^+^: 82% patient 2, 74% patient 3) (Figure 8B). On day 3 post-transfection, CAR expression was still above 50% on mRNA-CAR T cells derived from patients 2 and 3 and 26% on those from patient 1. Moreover, the resulting mRNA-CAR T cells derived from AML patients were shown to be more cytotoxic against CD123^+^ KG-1 cells for at least three days post-transfection, even at low effector-to-target cell ratios of 0.25:1 or 0.125:1 compared to the respective cytotoxicity of non-transfected T cells (-LNP) (Figure 8C), indicating mRNA-CAR T cell functionality.

## 3. Discussion

The optimal IVT-mRNA for non-viral CAR T cell generation should exhibit a high translation efficiency, a great stability, and minimal immunogenicity in order to result in a high CAR expression over the longest possible period of time. Here, the effect of different IVT-mRNA production and design strategies were investigated in human primary T cells, analyzing CAR expression as the main read-out parameter. As a starting point, the minimum components of an mRNA required for detectable CAR expression were determined to minimize the foreign RNA load and the associated negative effects [49]. The impact of further optimizations of this minimal RNA consisting only of a 5′-cap, a CAR-ORF, and a polyA-tail was then examined in detail step by step.

First, the most prominent and well-studied 5′-cap analogs, ARCA, and CleanCap were compared head-to-head in terms of mRNA production and in vitro performance. When using CleanCap as a cap analog, significant improvements were achieved compared to classical ARCA, including a higher transcription and capping efficiency as well as enhanced protein expression, which is in line with several previous reports [35,43,50,51,52]. In addition, using ARCA yielded less CAR-mRNA, which can be explained by the decreased GTP concentration in IVT reaction. However, the GTP reduction is required to achieve a high proportion of capped RNA, as ARCA and GTP compete with each other as the starting nucleotide [51]. Due to the trinucleotide structure of CleanCap, which shifts the equilibrium of the capping reaction to the side of the cap analog, a reduction in the GTP concentration is not necessary, and therefore better RNA capping and ultimately higher protein yields are achieved [51]. The capping efficiency was normalized to that of ARCA-RNA, because some measured values exceeded 1.00, which was reported before when LC-MS was used for determination [43,53], as in the present study. Thus, the precise capping efficiencies could not properly be determined. However, assuming an equal bias in every sample, the normalization of the measured values revealed an about 27% larger proportion of capped RNA when CleanCap was used compared to ARCA, which approximately corresponds to the data reported by Vlatkovik et al. [43]. There, 52–67% capping efficiency using ARCA and >90% using CleanCap were determined in a ribozyme-mediated cleavage assay combined with a silica-based column purification. In total, CleanCap outperformed ARCA capping in terms of the transcription, capping, and translation efficiency of CAR-mRNA, defining CleanCap as the most suitable reagent for CAR-mRNA generation. 

The beneficial effect of UTRs in IVT-mRNA on protein expression has been reported in multiple studies [54,55,56,57], establishing UTRs as an essential feature for therapeutic mRNAs. The hAG or human beta globin 5′ and 3′ UTRs are most widely used in mRNA-based therapeutics [23], whereby the hAG sequence was reported to outperform the hBG 5′ UTR regarding translation efficiency in living cells [58]. Orlandini von Niessen et al. [44] found the AES/mtRNR1 3′ UTR in combination with the hAG 5′ UTR to result in the highest IVT-mRNA half-life as well as reporter gene expression in human dendritic cells among a library of different 3′ UTRs. This can be attributed to a much lower number of potential binding sites for microRNAs [44] known to suppress mRNA translation and/or promote mRNA degradation [59], substantiating the usage of these UTRs in a slightly modified version in the Pfizer/BioNTech mRNA vaccine against SARS-CoV-2 (BNT162b2) [60]. Likewise, we observed an enhanced and prolonged CAR expression intensity in T cells for at least three days using CAR-mRNA with the hAG 5′ UTR and the AES/mtRNR1 3′ UTR. Besides the presence of binding sites for microRNAs in the 3′ UTR, protein expression can be influenced by several features of secondary structures within the 5′ UTR. For instance, the thermal stability of a hairpin and its distance from the 5′ cap were described to affect translation efficiency [58]. Babendure et al. [58] found that a hairpin with a thermal stability ranging from −10 to −25 kcal/mol at a position ranging from +7 to +13 enhance the translation of mRNA. The hAG 5′ UTR has been predicted to form a hairpin at the position +5 with a thermal stability of −8.4 kcal/mol [58], indicating only a weak support for an increased translation according to the mentioned study. Indeed, in another study, a synthetic UTR4 sequence resulted in a higher EGFP-mRNA translation in HeLa and immortalized murine bone marrow dendritic cells compared to the hAG 5′ UTR [56], suggesting that there might be further potential for 5′ UTR improvements for CAR-mRNA beyond the significant increase already shown here.

The modification of nucleotides, particularly m1ψ, has received special attention here regarding mRNA immunogenicity relative to protein expression in T cells, as it was previously reported to elicit a more pronounced activation of human primary macrophages compared to variations of the cap structure [35]. Irrespective of whether EGFP- or CAR-mRNA was used, m1ψ modification led to a reduction in protein expression to approximately 70% compared to no modification in primary T cells. These results agree with one former study [61]. However, the present results are also in striking contrast to those of several other studies reporting the superior protein expression of m1ψ-mRNA compared to unmodified mRNA in different adherent cell lines, including HEK293T cells, and primary keratinocytes [34], in murine cells of the monocytic lineage [62], in human mesenchymal stem cells [63], as well as in fibroblast-like synoviocytes [64]. In this study, an enhanced EGFP expression was likewise achieved with m1ψ-EGFP-mRNA in HEK293T cells, pointing to the cell-type dependent effects of m1ψ modification (Figure S1). 

Two main explanations for the improved protein expression of modified mRNA in cell-types other than T cells exist: an increased ribosome density along with a higher ribosomal recycling rate as well as a diminished translation inhibition or mRNA degradation [32,65]. Several mechanisms have been elucidated, explaining the reduced immunogenicity of nucleotide modifications and including (i) the lower binding affinity of modified mRNA to endosomal ssRNA sensors, TLR7 and TLR8 [30,33]; (ii) the decreased activation of cytosolic immune receptors, RIG-1 [33], as well as endosomal TLR3 [30], resulting in the lesser secretion of proinflammatory cytokines, and (iii) the reduction in unspecific promoter-independent anti-sense transcription by T7 RNA polymerase during IVT leading to fewer dsRNA molecules within the IVT-product [22]. This is in line with our results showing lower dsRNA quantities in m1ψ-modified mRNA and decreased TNF-α levels in the supernatant of transfected immune cells. However, despite the reduced immunogenicity, we observed that m1ψ led to lower protein levels in T cells, implying a different mechanism involved in translation modulation by nucleotide modifications in T cells. In fact, a decreased translational elongation rate was observed on modified mRNAs in a cell-free system [65]. A slower ribosome movement would negatively impact translation efficiency, but it was simultaneously shown, that this effect is compensated by an increased initiation rate, which is the major determinant of translation efficiency [65]. This leads to the speculation that, in T cells, there might be an imbalance between decelerating elongation and promoting initiation by m1ψ in IVT-mRNA. The modulation of the elongation rate could be the consequence of stabilized secondary structures of m1ψ-mRNA presenting a hurdle for translation [66], or altered interactions with mRNA-binding proteins [65]. The extent to which these suspected mechanisms play a role in T cells could be investigated in future studies. Mulroney et al. [67] recently reported that m1ψ in IVT-mRNA results in +1 ribosomal frameshifting and the occurrence of frameshifted polypeptides in HeLa cells present another reason for the reduced CAR expression levels caused by m1ψ-modified mRNA. Ribosomal frameshifting due to m1ψ was shown to require ribosome slippery sequences, which could be eliminated by codon optimizations [67]. Indeed, codon optimization regarding slippery sequences was not considered here and could be investigated in future studies. 

It has been shown that during IVT, when T7 RNAP is used, m1ψ is efficiently incorporated and does not disrupt the synthesis of full-length transcripts [68], which was also observed here. However, slightly higher error rates in m1ψ-RNA (7.4 ± 0.7 × 10^−5^ error/base) were reported compared to those in unmodified RNA (5.6 ± 0.8 × 10^−5^ error/base), mainly appearing as rA → rU substitutions [68]. Statistically, assuming a uniform error distribution, every ninth m1ψ-CAR-transcript of about 1500 nucleotides in length would have an error compared to every twelfth transcript having no nucleotide modification. Therefore, precise quality controls for mRNA identity need to be included when using m1ψ-CAR-mRNA for later clinical application. Altogether, this study shows that the m1ψ modification of mRNA does not necessarily result in improved translation in primary T cells. Nevertheless, we recommend m1ψ over unmodified mRNA, since it is less immunogenic, mainly attributed to less dsRNA contaminants, and more stable, which in the end balances the slightly impaired translation efficiency. 

In addition to mRNA structure and composition, the choice of IVT template as well as the IVT protocol used, especially the type of polymerase, had a major impact on the functionality of the CAR-mRNA. We could show that a fully optimized mRNA and IVT reaction balances the need for advanced downstream purification methods. DsRNA can efficiently be removed by HPLC or digestion by RNase III after IVT, but neither method was capable of completely evading the immune responses as shown here and by others [35,61,69]. More importantly, no significant reduction in immunogenicity was observed when using oligo-dT or HPLC purification in comparison to a simple spin column approach. Interestingly, spin-column approaches were sufficient for the purification of mRNA in a GMP-compliant protocol for ex vivo CAR T cell production [70]. These data indicate that a fully optimized mRNA and IVT reaction are of higher importance making more advanced downstream purification obsolete if the mRNA quality is already high enough for the desired application, such as ex vivo CAR T cell generation.

In summary, the CAR-mRNA design was stepwise optimized by the incorporation of CleanCap as 5′ cap analog, hAG 5′ UTR and AES/mtRNR1 3′ UTRs as well as m1ψ modification, which achieved a detectable CAR expression on cancer patients’ T cells for at least four days. We highlight the importance of a robust transcription protocol and its impact on the overall success of studies using mRNA-based transient CAR-T cells. Here, the most promising modifications which had led to advancements in previous studies have been combined showing an improved balance between translation efficiency in the T cells, stability, and immunogenicity of CAR-mRNA even in patient-derived T cells. Although mRNA-based CAR-T cells were tested here in a cancer-related setting, we believe that the results shown in our study are of even higher value for the field of autoimmune diseases, which requires a higher safety level of CAR-T cell products than the cancer indication, making transient CAR-T cells the favored approach here.

## 4. Materials and Methods

### 4.1. In Vitro Culture of Human Cell Lines

Semi-adherent HEK293T cells (ACC 635; DSMZ-German Collection of Microorganisms and Cell Cultures, Braunschweig, Germany) were cultured in Dulbecco’s Modified Eagle Medium (DMEM; #41966-029, Gibco^®^, ThermoFisher Scientific, Waltham, MA, USA) with 10% FBS (#10270-106, Gibco^®^, ThermoFisher Scientific, Waltham, MA, USA) and split in a 1:10 to 1:20 ratio every three to four days to maintain a cell confluency between 30% and 90%. Suspension cell line KG-1 (ACC 14; DSMZ-German Collection of Microorganisms and Cell Cultures, Braunschweig, Germany) was maintained at cell concentrations between 0.2 and 1.5 × 10^6^ cells/mL in Roswell Park Memorial Institute (RPMI) 1640 Medium (#21875-034, Gibco^®^, ThermoFisher Scientific, Waltham, MA, USA) with 10% FBS. Cell lines were grown at 37 °C and 5% CO_2_ and regularly checked for mycoplasma contamination. 

### 4.2. Isolation and In Vitro Culture of Primary Human T Cells

For T cell isolation, first, human peripheral blood mononuclear cells (PBMCs) were harvested from the blood of healthy donors (purchased by the Institute for Transfusion Medicine of University Clinic of Leipzig, Leipzig, Germany) or from cancer patients (collected at the Klinikum Chemnitz gGmbH, Chemnitz, Germany) using density gradient centrifugation with Ficoll-Paque PLUS (#17-1440-02, Th. Geyer GmbH & Co. KG, Renningen, Germany). Afterwards, T cells were isolated from PBMCs by magnetic-activated cell sorting using the Pan T Cell Isolation Kit (#130-096-535, Miltenyi Biotec, Bergisch Gladbach, Germany) according to the manufacturer’s instructions. For cultivation and expansion, T cell concentration was adjusted to 1 × 10^6^ cells/mL in ImmunoCult™-XF T Cell Expansion Medium (#10981, STEMCELL Technologies, Vancouver, BC, Canada) supplemented with 100 ng/mL human recombinant interleukin-2 (IL-2; #200-02, PeproTech, London, UK). T cells were stimulated by the addition of 25 µL/mL ImmunoCult™ Human CD3/CD28/CD2 T Cell Activator (#100-0785, STEMCELL Technologies, Vancouver, BC, Canada) and expanded at 37 °C and 5% CO_2_ until transfection on day 7 post-isolation.

### 4.3. Production of mRNA

Production of mRNA encoding a CD123-directed CAR construct (#CAR-LC028, Creative Biolabs, Shirley, NY, USA) started with the generation of the DNA template (PCR, plasmid linearization or PCR with digestion of the PCR product), followed by IVT with co-transcriptional 5′ capping. If no poly(A) tail was encoded in the DNA template, the polyadenylation of the 3′ RNA end was performed after IVT.

#### 4.3.1. Generation of the DNA Template

Since the DNA template for IVT requires a T7 promotor sequence upstream from the ORF, PCR from pDNA using forward primers with 5′ overhangs was performed to add the T7 promotor region where it was not already present in the pDNA. The standard T7 promotor sequence 5′-TAATACGACTCACTATA**GC**-3′ (bold GC: transcriptional start site) was incorporated to provide DNA templates for mRNA with the classical first-generation ARCA as 5′ cap structure, while 5′-TAATACGACTCACTATA**AG**-3′ (bold AG: transcriptional start site) was integrated in DNA templates for mRNA with a CleanCap AG (3′ OMe) (CleanCap) structure. For DNA template generation via PCR, 20 U/mL Q5 High-Fidelity DNA Polymerase (#M0491S, New England Biolabs, Ipswich, MA, USA) were used with 1× Q5 Reaction Buffer, 200 µM dNTPs (#5100850-0500, VWR, Darmstadt, Germany, 10 ng/mL pDNA used as template for PCR, each 0.5 µM respective forward and reverse primer, and 1× Q5 High GC Enhancer according to manufacturer’s instructions. Reactions were incubated for 30 s at 98 °C (initial denaturation), followed by 35 cycles of 10 s at 98 °C (denaturation) and 90 s at 72 °C (annealing and extension), followed by 2 min at 72 °C (final extension). Optionally, the obtained PCR product was digested with 10 units of BspQI (#R0712L, New England Biolabs, Ipswich, MA, USA) per 1 µg DNA. To produce the DNA template for IVT using pDNA with an integrated T7 promotor region, the pDNA was also linearized with 10 units of BspQI per 1 µg DNA. The PCR and digestion samples were purified using the NucleoSpin Gel and PCR Clean-up Mini kit (#740609.250, MACHEREY-NAGEL, Düren, Germany). The concentration of the DNA templates was determined using UV/Vis-spectroscopy at NanoDrop 3000 (ThermoFisher Scientific, Waltham, MA, USA). Integrity was analyzed by agarose gel electrophoresis. Purified DNA templates were stored at −20 °C until IVT was performed.

#### 4.3.2. In Vitro Transcription

Originating from the DNA template with the T7 promotor sequence, RNA was synthesized by IVT with co-transcriptional 5′ capping using the HighYield T7 RNA Synthesis Kit (#RNT-101, Jena Bioscience, Jena, Germany) or the HiCap RNA polymerase (#E014-B032A, Codexis, Redwood City, CA, USA). The comparison of IVT with different cap analogs was performed using the HighYield T7 RNA Synthesis Kit following the respective recommendations of the cap analog manufacturers. As such, IVT reactions were assembled to reach 1× HighYield T7 Reaction Buffer, 10 mM DTT, either 6 mM ARCA (#NU-855L, Jena Bioscience, Jena, Germany) or 5 mM CleanCap Reagent AG (3′ OMe) (#040N-7413-1, TriLink Biotechnologies, San Diego, CA, USA), 7.5 mM ATP (ARCA) or 5 mM ATP (CleanCap), 7.5 mM CTP (ARCA) or 5 mM CTP (CleanCap), 7.5 mM UTP/N^1^-methylpseudo-UTP (Jena Bioscience, Jena, Germany) (ARCA) or 5 mM UTP/N^1^-methylpseudo-UTP (CleanCap), 1.5 mM GTP (ARCA) or 5 mM GTP (CleanCap), 1 µg template DNA, and 10% (*v*/*v*) HighYield T7 RNA Polymerase Mix. IVT reactions for the HiCap RNA polymerase were prepared according to the manufacturer’s recommendations and contained 1× Codex PDHC3 Reaction Buffer, 3 mM DTT, 1.5 mM CleanCap Reagent AG (3′ OMe), 5 mM ATP, 5 mM CTP, 5 mM UTP/N^1^-methylpseudo-UTP, 5 mM GTP, 1 µg template DNA, 2 mU/µL Yeast Thermostable Inorganic Pyrophosphatase (#M0296S, New England Biolabs, Ipswich, MA, USA), 1 U/µL RNase Inhibitor (#N2515, Promega Corporation, Madison, WI, USA) and 2.5% (*v*/*v*) HiCap RNA polymerase solution. All IVT reactions were incubated for up to 4 h at 37 °C. Products were subsequently treated with 100 U/mL TURBO™ DNase (#AM2238, ThermoFisher Scientific, Waltham, MA, USA) for 15 min at 37 °C to eliminate the DNA template. 

The IVT products were standardly purified using the NucleoSpin RNA Clean-up Mini kit (#740948.50, MACHEREY-NAGEL, Düren, Germany). In the mRNA purification experiments, IVT products were purified using the Magnetic mRNA Isolation Kit with Oligo d(T)_25_ Magnetic Beads (#S1550s, New England Biolabs, Ipswich, MA, USA) or by HPLC Agilent PLRP-S column (Agilent Technologies, Santa Clara, CA, USA) 100 mM TEAA as running buffer, gradient elution to max. 25% (*v*/*v*) CH3CN. Concentration of purified mRNA was determined at NanoDrop 3000 or NanoDrop One. The RNA was analyzed using agarose gel electrophoresis supplemented with 0.3% hydrogen peroxide (#9681.4, Carl Roth, Karlsruhe, Germany) or with a 2100 Bioanalyzer instrument (Agilent Technologies, Santa Clara, CA, USA). Capping efficiency of ARCA- and CleanCap-RNA was determined by Tamaserv UG (Mainz, Germany) using liquid chromatography–mass spectrometry (LC-MS). Purified samples were stored at −80 °C until usage or polyadenylation was conducted.

For the addition of a 3′ poly(A) tail to untailed RNA, 25 U/mL *E. coli* Poly(A) Polymerase (#M0276L, New England Biolabs, Ipswich, MA, USA) was used with 450 ng/µL IVT product, 1× *E. coli* Poly(A) Polymerase Reaction Buffer, 1 mM ATP, and 5 U/µL RNasin RNase inhibitor (#N2515, Promega Corporation, Madison, WI, USA) according to the manufacturer’s instructions. Reactions were incubated for 1 h at 37 °C and immediately purified via the NucleoSpin RNA Clean-up Mini kit (#740948.50, MACHEREY-NAGEL, Düren, Germany). Analysis via NanoDrop and agarose gel electrophoresis were performed as after IVT. Samples were stored at −80 °C until usage.

### 4.4. Cell-Based mRNA Expression Analysis

For simple and fast translation analysis in a cell-based system, HEK293T cells were transfected with produced IVT-mRNA. Therefore, 1 × 10^5^ HEK293T cells in 500 µL DMEM + 10% FBS medium were plated in a 24-well plate and pre-cultured for 4 h at 37 °C and 5% CO_2_ to allow attachment of the cells to the bottom plate. Afterwards, the cells were transfected with 1 pmol of mRNA using Lipofectamine 2000 Transfection Reagent (#11668027, ThermoFisher Scientific, Waltham, MA, USA) with Opti-MEM reduced serum medium (#31985062, Gibco^®^, ThermoFisher Scientific, Waltham, MA, USA) according to the manufacturer’s protocol. After 24 h, transfected cells were harvested and analyzed regarding CAR expression by flow cytometry.

### 4.5. Transfection of T Cells with mRNA

For the delivery of generated mRNA into T cells, isolated and expanded T cells were either transfected with mRNA by electroporation or by mRNA-loaded lipid nanoparticles (mRNA-LNPs).

#### 4.5.1. Electroporation

For mRNA delivery into T cells, electroporation was performed using the Neon Transfection System 10 μL Kit (#MPK1025, ThermoFisher Scientific, Waltham, MA, USA) according to the manufacturer’s protocol. Therefore, T cells were harvested on day seven post-isolation, washed in 1x PBS, and subsequently resuspended in buffer R (supplied with the kit). Then, 1 × 10^6^ cells were mixed with 6 µg of mRNA. Cell concentration was adjusted to 2 × 10^7^ cells/mL by the addition of buffer R and electroporated at 1600 V, 10 ms, 3 pulses (4 × 10 µL). Electroporated cells were transferred in pre-warmed T cell expansion medium with IL-2 to reach a cell concentration of 5 × 10^5^ cells/mL. Cells were incubated at 37 °C and 5% CO_2_ until further analyses were performed. 

#### 4.5.2. mRNA-LNP Formulation and T Cell Transfection

As an alternative mRNA delivery method, mRNA-LNPs were formulated using the GenVoy-ILM T Cell Kit for mRNA (#1000701, Cytiva, now including Precision NanoSystems, Marlborough, MA, USA), and incubated with T cells as previously described by us in detail [46].

### 4.6. Flow Cytometry

CAR expression of the transfected T cells was flow cytometrically analyzed on BD FACSCanto^TM^ II (BD Biosciences, Mississauga, ON, Canada) or on MACSQuant10 (Miltenyi Biotec, Bergisch Gladbach, Germany) using PE or AlexaFluor 647-conjugated AffiniPure polyclonal goat F(ab’)₂ Anti-Mouse IgG, F(ab’)₂ fragment specific with minimal cross-reactions to human, bovine, and horse serum proteins (#JIM-005-600-006, Jackson ImmunoResearch, Ely, UK) or APC-coupled anti-DYKDDDDK (FLAG) Tag Antibody (clone: L5) (#637307, BioLegend, San Diego, CA, USA). Ten minutes prior to measurement, 7-aminoactinomycin D (7-AAD, #559925, BD Biosciences, Mississauga, Canada) was added to stain dead cells. Recorded events were gated for single cells (forward scatter-height (FSC-H) x FSC-area (FSC-A)), then for viable cells either gated via FSC-A x sideward scatter-area (SSC-A) (life gate) or via 7-AAD-negative signals. The data were evaluated in Kaluza Analysis software (version 2.1; Beckman Coulter, Krefeld, Germany).

### 4.7. Quantitative-Reverse Transcriptase-PCR (q-RT-PCR)

To determine transferred CAR-mRNA quantity inside cells, 24 h after transfection cells were harvested and washed in 1× PBS. Cell pellets were resuspended in TRIzol reagent (#15596026, Zymo Research, Freiburg, Germany) and frozen at −80 °C until RNA isolation was performed using the Direct-zol RNA Microprep Kit according to manufacturer’s instructions. The concentration of the isolated RNA was determined at NanoDrop 2000c. Reverse transcription of 100 ng isolated RNA was performed using RevertAid First Strand cDNA Synthesis Kit (#K1622, ThermoFisher Scientific, Waltham, MA, USA) with 1× reaction buffer, 2 U/µL RiboLock RNase Inhibitor, 10 mM dNTP Mix, 20 U/µL RevertAid M-MuLV Reverse Transcriptase, and 10 µM Oligo d(T)_18_ primer. The mixture was incubated for 60 min at 42 °C followed by 5 min at 70 °C. The resulting cDNA was diluted 1:1000 in nuclease-free H_2_O. For quantitative PCR, 5 µL of PerfeCTa SYBR Green FastMix reagent (#733-1381, Quantabio, Beverly, MA, USA) was mixed with 1 µL diluted cDNA and each 0.5 µL of the CAR-specific primer pair. Reaction mixes were incubated for 10 min at 95 °C, followed by 40 cycles of 10 s at 95 °C and 30 s at 64 °C (annealing and extension), followed by a standard melting curve analysis on the qPCR cycler (LightCycler 480, Roche, Rotkreuz, Switzerland). GAPDH was used as housekeeping gene.

### 4.8. RNA Immunogenicity Assay

To analyze the immunogenicity of produced IVT-mRNA, human primary PBMCs were transfected with IVT-mRNA and analyzed 24 h later for their T cell activation and TNF-α secretion. For the mRNA transfection of PBMCs, 2 µL of Lipofectamine MessengerMAX Transfection Reagent (#LMRNA008, ThermoFisher Scientific, Waltham, MA, USA) was diluted in 50 µL Opti-MEM medium and incubated for 10 min at RT. In the meantime, 1 µg of IVT-mRNA was diluted in 50 µL Opti-MEM, which was added at a 1:1 ratio to the diluted Lipofectamine MessengerMAX solution. This mixture was incubated for 5 min at RT and 25 µL (corresponding to 250 ng mRNA) was added to a well of a 24 F-bottom well plate, immediately followed by the addition of 5 × 10^5^ PBMCs in 500 µL RPMI + 10% FBS medium. As positive controls for immune stimulation, equivalent amounts of dsRNA (#RNT-SCI-10080100, Jena Bioscience, Jena, Germany) and Resiquimod (R-848, # SML0196-10MG, Merck KGaA, Darmstadt, Germany) were used. The cells were incubated at 37 °C and 5% CO_2_. After 24 h, T cell activation was analyzed by flow cytometry using anti-CD3–FITC (clone: Sk7, #555332) and anti-CD69–APC-H7 (clone: FN50, # 557756) antibodies (BD Biosciences, Mississauga, Canada). For TNF-α detection, the supernatant of transfected PBMCs was collected and stored at −80 °C until analysis via enzyme-linked immunosorbent assay (ELISA). Therefore, the supernatant was thawed, diluted 1:10 in distilled H_2_O and then analyzed using the TNF alpha Human Uncoated ELISA Kit with Plates (#88-7346-22, ThermoFisher Scientific, Waltham, MA, USA) according to manufacturer’s instructions. 

### 4.9. Dot Blot

For the quantification of the dsRNA content of produced IVT-mRNAs, a dot plot was performed according to the protocol previously described by Moradian et al. [35] with slight adaptations. Briefly, 1000 ng of IVT-mRNA samples were blotted on a Nytran supercharged blotting membrane (#10416294, VWR, Darmstadt, Germany) using a 96-well dot plot apparatus (Bio-Rad Laboratories GmbH, Feldkirchen, Germany). In parallel, 1:4 serial dilutions of dsRNA used as standard were blotted on the same membrane, starting at 1000 ng as the highest amount. The equal volume of nuclease-free H_2_O was applied as negative control. After sample loading, the membrane was air-dried for at least 30 min and blocked using blocking buffer (5% (*w*/*v*) skim milk powder in 1x Tris-buffered saline with 0.1% (*v*/*v*) Tween 20 (TBS-T) buffer) for 1 h at room temperature. The membrane was incubated in 20 mL of 1:1000 diluted dsRNA-directed antibody (clone: K1) (Jena Bioscience, Jena, Germany) in antibody buffer (1% (*w*/*v*) skim milk powder in 1× TBS-T buffer) at 4 °C overnight on a plate shaker. The membrane was washed three times in 1× TBS-T buffer for 5 min each, and incubated in 20 mL of 1:2000 diluted horseradish peroxidase (HRP)-conjugated anti-mouse IgGκ (#sc-516102, Santa Cruz Biotechnology, Inc., Dallas, TX, USA) in antibody buffer for 1 h at room temperature. After washing three times in 1× TBS-T buffer for 15 min each, the membrane was treated with 1 mL enhanced chemiluminescence (ECL) western blotting substrate (#32109, ThermoFisher Scientific, Waltham, MA, USA) and immediately analyzed using a chemiluminescence detector system (Intas Science Imaging Instruments GmbH, Göttingen, Germany). Signal intensities were densitometrically quantified using ImageJ software version 1.53t (National Institutes of Health, Bethesda, MD, USA) and the dsRNA amount was determined using a dsRNA standard curve.

### 4.10. CAR T Cell Cytotoxicity Assay

To analyze the functionality of the mRNA-CAR T cells deriving from cancer patients, KG-1 target cells were labeled using CellTrace™ Violet Cell Proliferation Kit, for flow cytometry (#C34557, ThermoFisher Scientific, Waltham, MA, USA) according to the manufacturer’s instructions. Subsequently, 25,000 of the labeled target cells were plated in RPMI + 10% FBS medium in a 96 U-bottom well plate together with different numbers of CAR T effector cells to achieve various effector-to-target (E:T) ratios. Irrespective of the proportion of CAR^+^ cells upon T cell transfection, the whole T cell population was considered as effector cells. Target cells incubated in RPMI + 10% FBS without effector cells were used as a reference sample, and target cells treated with 0.1% Tween 20 were used as a positive control that indicates 100% cytotoxicity. The cells in the plate were centrifuged at 120× *g* for 2 min and cultured at 37 °C and 5% CO_2_ for 24 h. After incubation, the cells were harvested, incubated with 7-AAD, and analyzed on BD FACSCanto^TM^ II. Cytotoxicity was calculated with the following formula: cytotoxicity [%] = 100% − (CellTrace Violet^+^7-AAD^−^ cells in co-culture sample [%]/CellTrace Violet^+^7-AAD^−^ cells in the reference sample [%]) × 100%. 

### 4.11. Statistics

The generation of the graphs and statistical analysis were performed using GraphPad Prism version 6.07 for Windows (GraphPad Software, Inc., La Jolla, CA, USA). Values are presented as the mean ± standard error of the mean (SEM) or standard deviation (SD) as specified in the figure legends. To determine significant differences between the means of two unmatched groups, datasets were tested for Gaussian distribution using the Shapiro–Wilk test. For normally distributed data, an F test was applied to compare variances. For non-significantly different variances (*p* > 0.05), an unpaired *t*-test was performed. If significantly different variances (*p* < 0.05) were determined, an unpaired *t*-test with Welch’s correction was performed. When two groups with data that were obtained using cells from the same blood donor were compared, groups were considered as matched. In this case, the Shapiro–Wilk test was performed to test whether the differences of the pairs follow a Gaussian distribution. For normally distributed differences, a paired *t*-test was performed. To determine significant differences between the means of more than two unmatched groups, either one-way analysis of variance (ANOVA) was performed in the case of normally distributed data, or the Kruskal–Wallis test was applied in the case of non-normally distributed data. P-values were adjusted for multiple comparisons by Dunn’s or Holm–Sidak multiple comparison test where applicable and are symbolized by asterisks as follows: * for *p* < 0.05, ** for *p* < 0.01, *** for *p* < 0.001, and **** for *p* < 0.0001. 

## 5. Conclusions

In summary, the CAR-mRNA design was optimized by the incorporation of CleanCap as 5′ cap analog, hAG 5′ UTR, and AES/mtRNR1 3′ UTRs as well as m1ψ modification. The extent to which this design represents the optimal one cannot be ascertained, as not all possible modifications have been tested. Here, the most promising modifications which led to advancements in previous studies have been selected showing an improved balance between translation efficiency in the T cells, stability, and immunogenicity of CAR-mRNA. In addition, this optimized CAR-mRNA was successfully used in vitro for CAR-mediated killing by transient CAR T cells from AML patient samples.

## Data Availability

Data are available from the corresponding author upon reasonable request.

## Supplementary Materials

The following supporting information can be downloaded at: https://www.mdpi.com/article/10.3390/ijms26030965/s1.
