# Supplementary material for: Optimal Chimeric Antigen Receptor (CAR)-mRNA for Transient CAR T Cell Generation
Source: Int J Mol Sci. 2025 Jan 23;26(3):965. doi: 10.3390/ijms26030965 (PMC11818003; doi:10.3390/ijms26030965)
Supplement: Supplementary file 1 [file ijms-26-00965-s001.zip › ijms-3410516-supplementary.pdf]

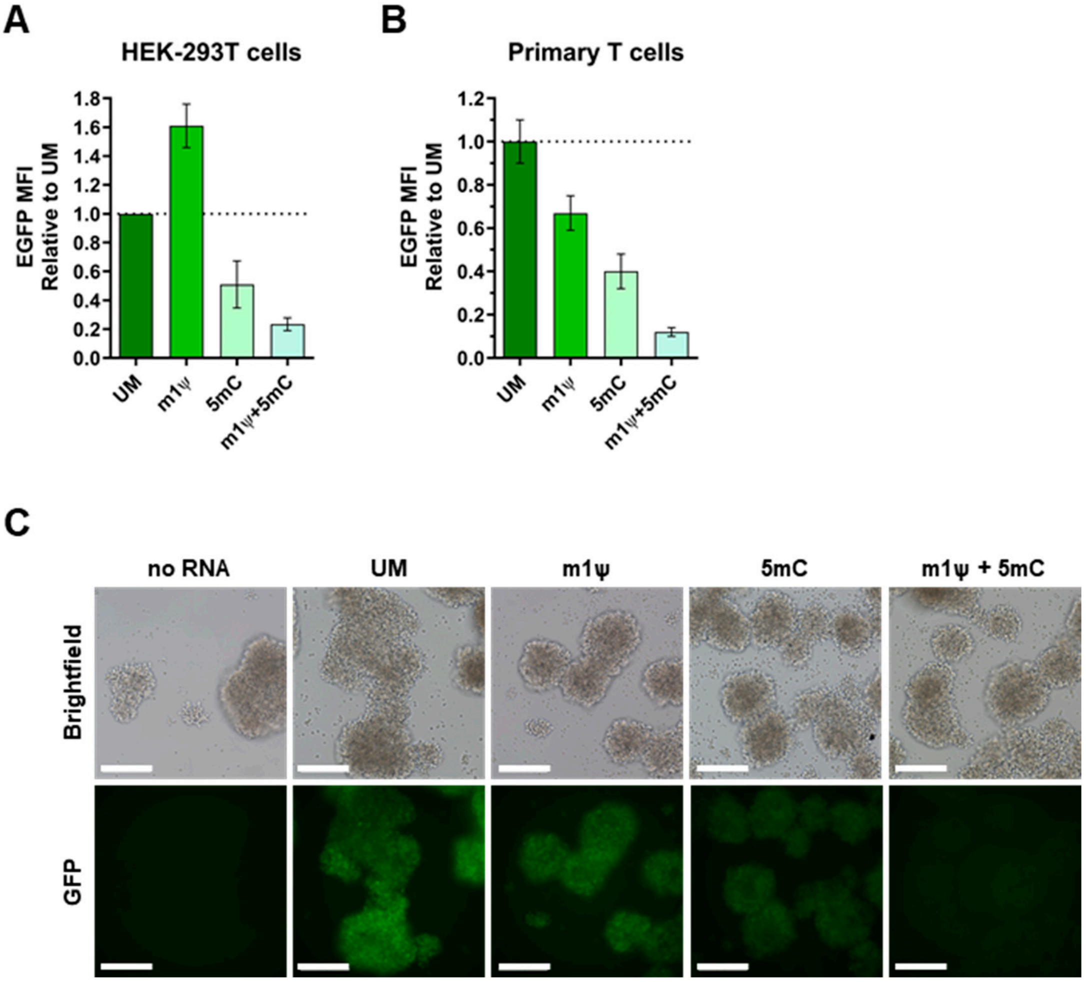

Figure S1: Translation of enhanced green fluorescent protein (EGFP)-mRNA with modified nucleotides. Uridine was replaced by N^1-methlypseudouridine (m1ψ), and/or cytidine was substituted by 5-methylcytidine (5mC). **(A, B)** EGFP median fluorescence intensity (MFI) in (A) HEK-293T cells and (B) in primary human T cells transfected with unmodified (UM) or modified mRNA as indicated, determined by flow cytometry 24 h after transfection. **(C)** Representative light (top) and fluorescence microscopic images (bottom) of T cells 24 h after transfection via lipid nanoparticles with no, UM or modified mRNA as indicated. (A, B) Columns indicate mean ± SEM of n=2 independent experiments (A) performed in technical triplicates or (B) with T cells from two different donors. (C) Scale bar indicates 200 μm.

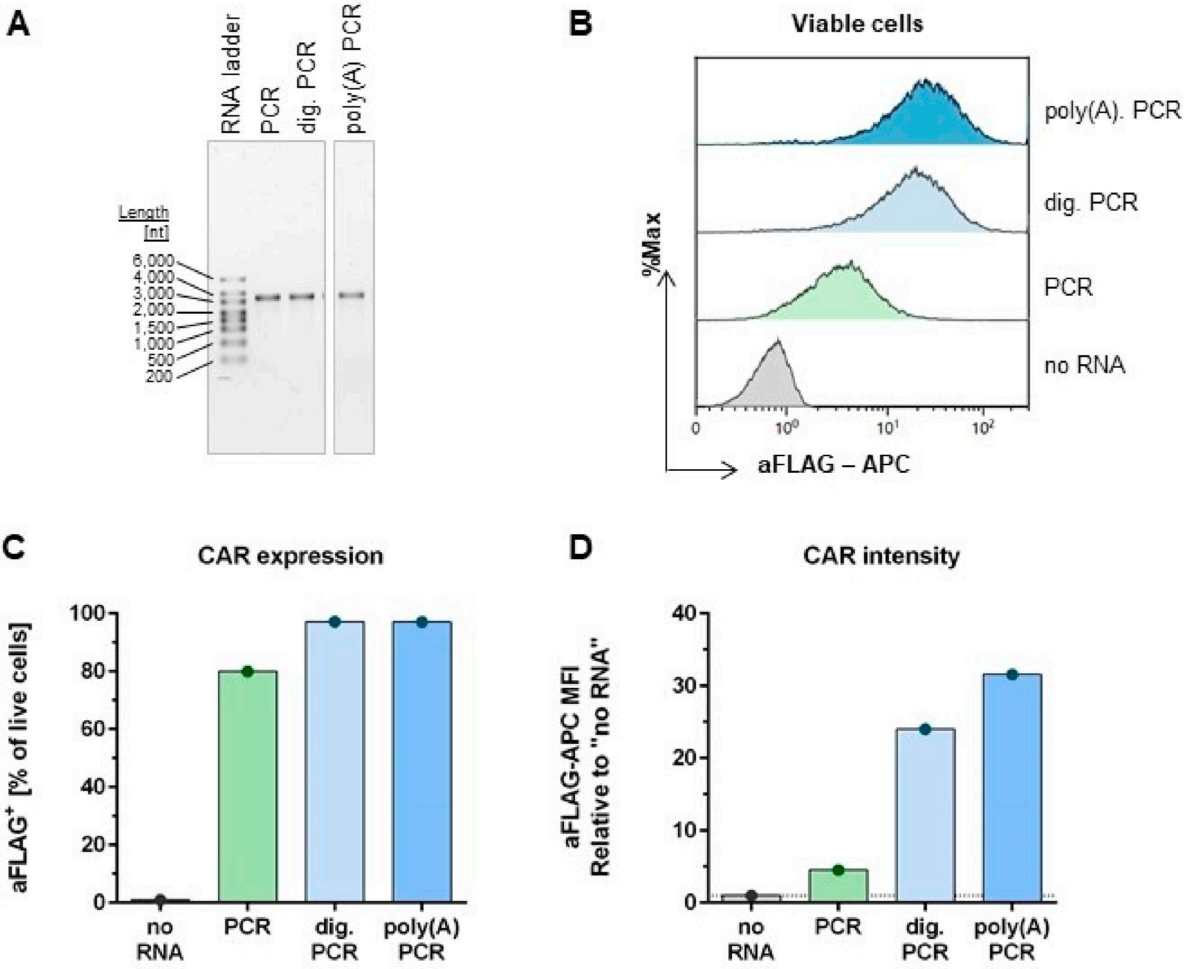

Figure S2: Optimized PCR product improves CAR expression. (A) Representative agarose gel image of CAR-mRNAs produced from PCR product, digested PCR product and poly(A) PCR product. A poly(A) tail of 120 nt length was attached downstream of the 3′ UTR via primer overhang. All samples were run on the same gel. Image was modified to remove irrelevant lanes. **(B)** Representative histograms of flow cytometric CAR expression analysis of viable Jurkat cells 24 h after electroporation with CAR-mRNA produced from different PCR templates. **(C)** Percentage of CAR^{+} Jurkat cells 24 h after electroporation. **(D)** CAR intensity on cell surface of Jurkat cells 24 h after transfection relative to cells that received no mRNA. (C-D) Bars indicate data from one electroporation.

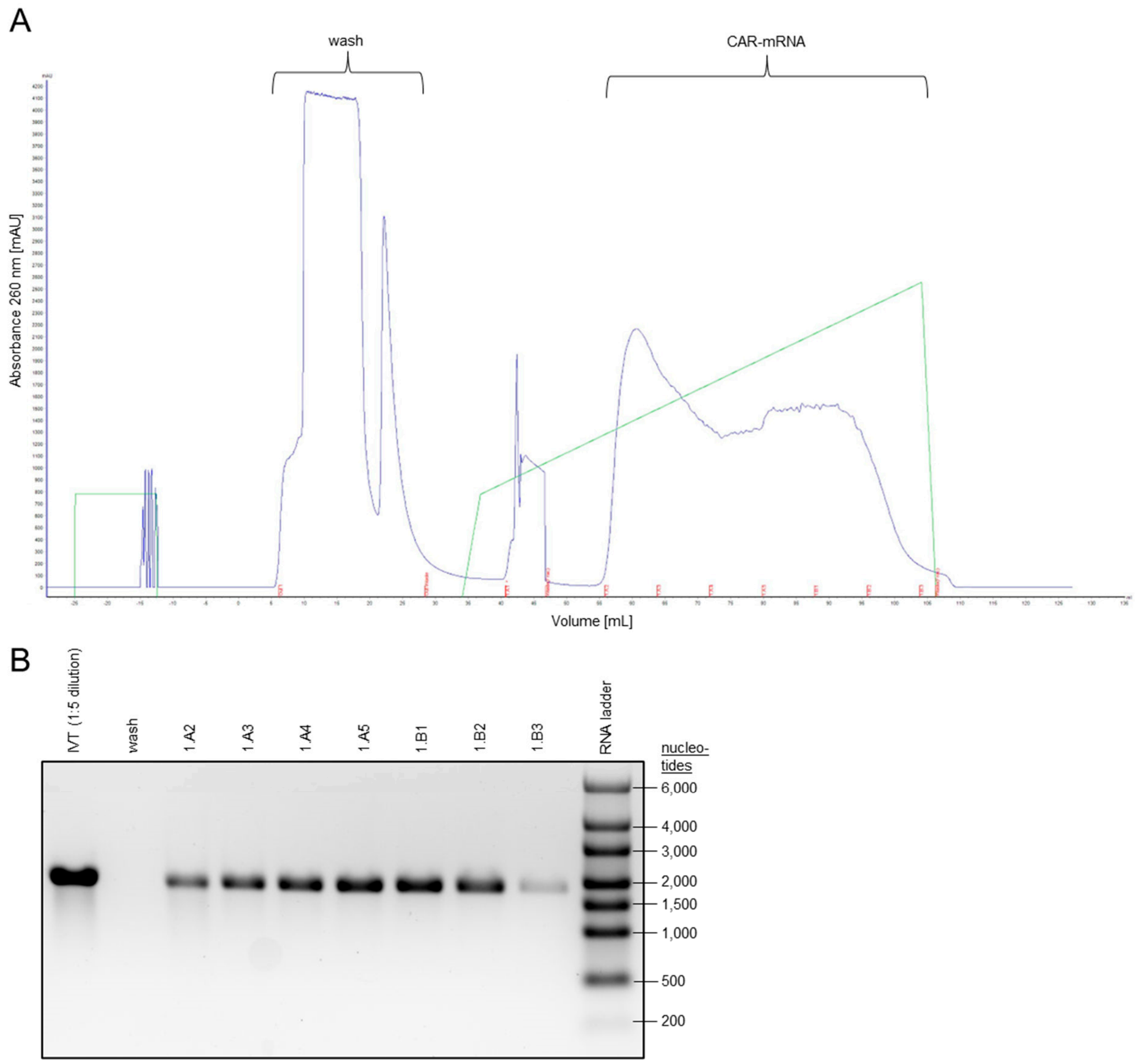

Figure S3: Purification of CAR-mRNA using HPLC. (A) Representative chromatogram of CAR-mRNA purification using HPLC (reversed-phase liquid chromatography). Column size 4,15 ml. Blue: absorbance at 260 nm, green: concentration eluent (25 %(v/v) acetonitrile). **(B)** Representative agarose gel image of purified CAR-mRNA fractions after elution from the column.

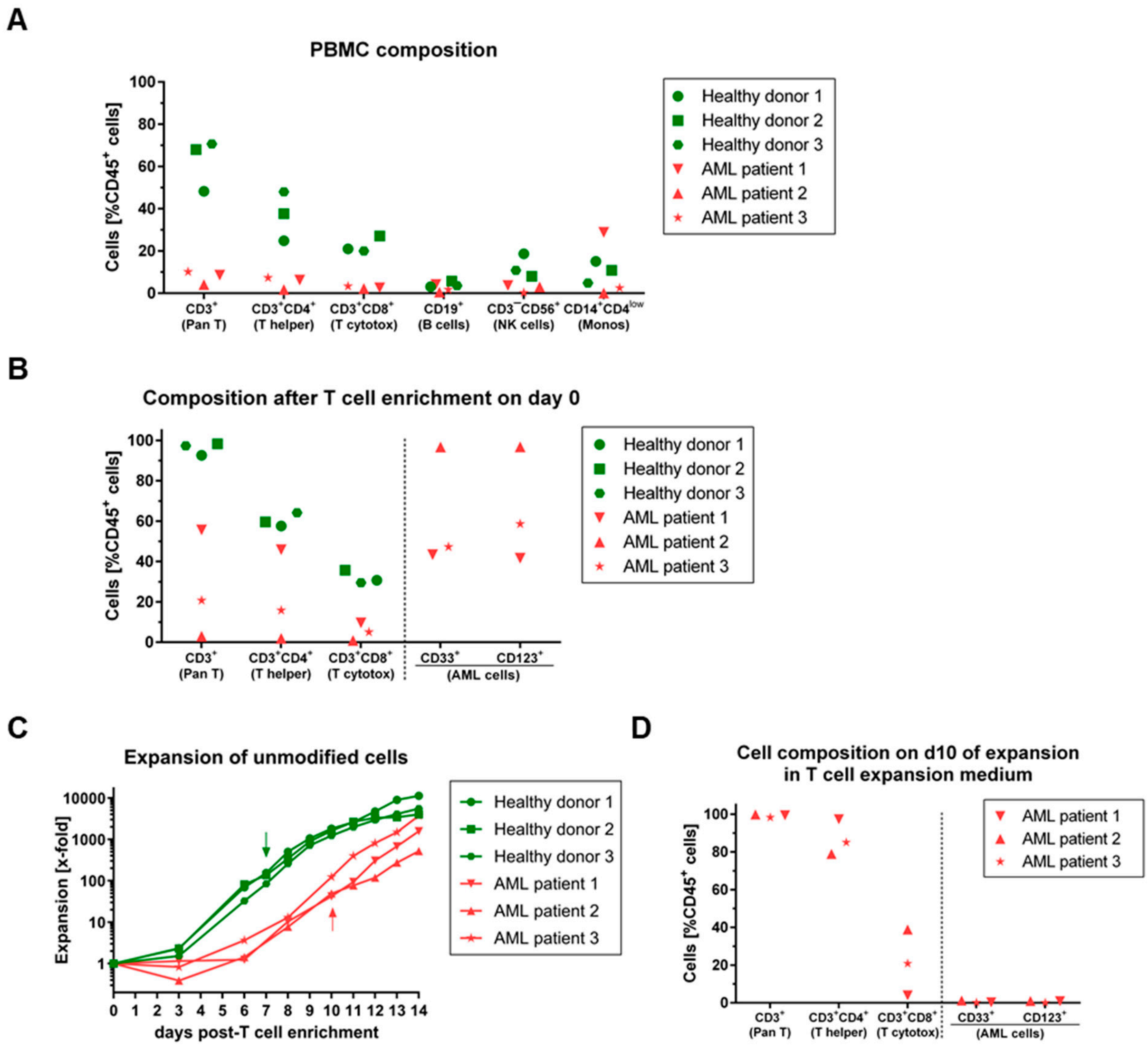

Figure S4: Preparation of T cells from acute myeloid leukemia (AML) patients. (A) Peripheral blood mononuclear cells (PBMC) composition prior T cell enrichment in comparison to that of healthy blood. The whole T cell population (Pan T) with two subtypes, helper T cells (T helper) and cytotoxic T cells (T cytotox), as well as B cells, natural killer (NK) cells and monocytes (Monos) were quantified by flow cytometry. **(B)** Composition of cells after CD3$^+$ T cell enrichment. Besides T cells and subtypes, two AML markers, CD33 and CD123, were quantified for cells from AML patients. **(C)** Expansion of unmodified cells after T cell enrichment and activation. Arrows indicate the selected time point of mRNA-LNP addition to the cells (green downwards: healthy donors, red upwards: AML patients). **(D)** Composition of T cells on day 10 of cell expansion to determine T cell populations and AML cells.
